# DCMC-UNet: A Novel Segmentation Model for Carbon Traces in Oil-Immersed Transformers Improved with Dynamic Feature Fusion and Adaptive Illumination Enhancement

**DOI:** 10.3390/s25133904

**Published:** 2025-06-23

**Authors:** Hongxin Ji, Jiaqi Li, Zhennan Shi, Zijian Tang, Xinghua Liu, Peilin Han

**Affiliations:** 1 School of Electrical Engineering, China University of Mining and Technology, Xuzhou 221116, China; ts23230113p31@cumt.edu.cn (J.L.); ts24230126p31@cumt.edu.cn (Z.S.); ts23230145p31@cumt.edu.cn (Z.T.); ts22230107p31@cumt.edu.cn (P.H.); 2 College of Mechanical and Electronic Engineering, Shandong Agricultural University, Tai’an 271018, China; lxh9357@163.com

**Keywords:** transformer inspection robot, semantic segmentation, U-Net, adaptive illumination enhancement, dynamic feature fusion

## Abstract

For large oil-immersed transformers, their metal-enclosed structure poses significant challenges for direct visual inspection of internal defects. To ensure the effective detection of internal insulation defects, this study employs a self-developed micro-robot for internal visual inspection. Given the substantial morphological and dimensional variations of target defects (e.g., carbon traces produced by surface discharge inside the transformer), the intelligent and efficient extraction of carbon trace features from complex backgrounds becomes critical for robotic inspection. To address these challenges, we propose the DCMC-UNet, a semantic segmentation model for carbon traces containing adaptive illumination enhancement and dynamic feature fusion. For blurred carbon trace images caused by unstable light reflection and illumination in transformer oil, an improved CLAHE algorithm is developed, incorporating learnable parameters to balance luminance and contrast while enhancing edge features of carbon traces. To handle the morphological diversity and edge complexity of carbon traces, a dynamic deformable encoder (DDE) was integrated into the encoder, leveraging deformable convolutional kernels to improve carbon trace feature extraction. An edge-aware decoder (EAD) was integrated into the decoder, which extracts edge details from predicted segmentation maps and fuses them with encoded features to enrich edge features. To mitigate the semantic gap between the encoder and the decoder, we replace the standard skip connection with a cross-level attention connection fusion layer (CLFC), enhancing the multi-scale fusion of morphological and edge features. Furthermore, a multi-scale atrous feature aggregation module (MAFA) is designed in the neck to enhance the integration of deep semantic and shallow visual features, improving multi-dimensional feature fusion. Experimental results demonstrate that DCMC-UNet outperforms U-Net, U-Net++, and other benchmarks in carbon trace segmentation. For the transformer carbon trace dataset, it achieves better segmentation than the baseline U-Net, with an improved mIoU of 14.04%, Dice of 10.87%, pixel accuracy (P) of 10.97%, and overall accuracy (Acc) of 5.77%. The proposed model provides reliable technical support for surface discharge intensity assessment and insulation condition evaluation in oil-immersed transformers.

## 1. Introduction

As critical pieces of equipment in power systems, large oil-immersed transformers play a vital role in grid operation. By assessing the operational status of large transformers, preventive measures can be implemented in advance to effectively avoid major power accidents, thereby ensuring the safe and stable operation of power systems. However, due to their hermetical-sealed metal construction, the internal inspection of large power transformers cannot be performed without disassembly. If solely relying on dissolved gas analysis (such as the three-ratio method [1,2] and gas chromatography [3,4]), one cannot accurately locate defects, determine their types, or assess severity levels. Currently, four major challenges exist in internal insulation detection for large power transformers: The first is low detection efficiency. Traditional inspection methods require either manual entry into the transformer body or removal of the tank cover, involving complex procedures such as oil drainage, refilling, and filtration. These take at least 15 days while causing prolonged power outages. The second challenge is insufficient accuracy. The complex internal structure and confined space make manual inspection inadequate for precisely identifying defect types, locations, and severity levels, resulting in non-targeted maintenance strategies. The third challenge is safety hazards. Personnel entering the confined space may collide with internal components, endangering both the personnel and the equipment’s safety. The fourth challenge is poor cost-effectiveness. Inaccurate assessment of defect types and severity levels may lead to unnecessary transportation to repair facilities for oil extraction, hoisting, and disassembly operations, incurring substantial transportation, cover removal, and maintenance costs.

Featuring a compact size and high mobility, inspection micro-robots demonstrate distinct advantages for visual inspection in transformers, enabling the direct localization of insulation faults and assessment of insulation degradation. Consequently, many research institutions have initiated the development of inspection micro-robots for transformers. In 2018, ABB launched Txplore [5], the first oil-immersed transformer inspection robot, whose innovative design earned it the 2019 Electrical Review Award (UK). Shenyang Ligong University adapted underwater robotics technology to develop SSTIR, a spherical inspection robot with a diameter of 19 cm, equipped with two vertical oil jet thrusters [6]. Due to its slight positive buoyancy, the vertical thrusters provide propulsion only during descent, while the horizontal thrusters control forward motion and steering, with cameras mounted on the robot’s mid-section. Furthermore, Tsinghua University, China University of Mining and Technology, and the Tianjin Electric Power Research Institute collaboratively developed an intelligent robotic fish for internal transformer inspection, examining its application advantages and feasibility while conducting in-depth research on attitude positioning, defect recognition, and path planning [7,8,9,10,11].

During their operation inside transformers, current inspection robots continuously capture internal environment images through visual cameras and transmit them via wireless antennae to computer workstations, relying on the manual identification of target defects. However, given the massive volume and complex internal structures of large transformers, sustained human observation not only imposes heavy workloads but may also lead to the missed detection of certain defects. Consequently, when employing micro-robots for the internal visual inspection of large transformers, addressing challenges such as extensive inspection ranges and significant variations in defect morphology (e.g., surface discharge carbon traces) and dimensions, the intelligent and efficient extraction of defect targets from complex backgrounds becomes crucial for accomplishing robotic inspection tasks for transformers.

Deep-learning-based object detection algorithms [12,13,14] have emerged as effective solutions for carbon trace image recognition and classification, leveraging their strong adaptability, low dependency on background models, and robust feature extraction capabilities, combined with real-time processing advantages. As one of the core tasks in computer vision, semantic segmentation has also achieved significant progress in recent years. Deep-learning segmentation methods are primarily categorized into two-stage and single-stage semantic segmentation approaches. Two-stage segmentation methods include both detection-based and segmentation-based paradigms. Detection-based algorithms (e.g., Mask R-CNN [13]) first generate candidate regions through a detector, followed by producing pixel-level masks for each instance. While achieving high accuracy, these algorithms suffer from slow processing speeds, complex model architectures, high computational demands, and challenging training processes, making them difficult to deploy on low-performance devices. In contrast, segmentation-based two-stage algorithms omit the detection step, performing direct pixel-level semantic segmentation followed by mask generation through pixel clustering. However, this approach demonstrates limited segmentation accuracy and generalization capability due to its excessive reliance on mask annotations. Single-stage segmentation methods (e.g., YOLACT [14], SOLO [15]) simultaneously accomplish classification, detection, and mask generation, offering high frame rates and compact model sizes that facilitate both training and engineering deployment, making them ideal candidates for algorithmic improvements in semantic segmentation. Fully Convolutional Networks (FCNs) and their variants, particularly U-Net, have become mainstream technologies. Proposed by Long et al., FCNs achieve end-to-end pixel-level classification by replacing fully connected layers with convolutional layers, establishing the foundation for image segmentation [16]. U-Net [17], introduced by Ronneberger et al. in 2015 for medical image segmentation, enhances FCNs by incorporating skip connections that fuse shallow and deep features, significantly improving accuracy and demonstrating exceptional performance in medical applications. The U-Net architecture has continuously evolved, spawning numerous variants including Attention-UNet [18] and U2Net [19].

While U-Net and its derivatives perform well in segmenting images with regular morphology and low edge curvature (e.g., medical imaging and traffic scenarios), they face notable challenges in transformer discharge carbon trace segmentation. During surface discharge in transformers, the spatial randomness of arc ablation and uncertainty in discharge intensity produce carbon traces characterized by complex edge structures, significant morphological diversity, and large dimensional variations. These characteristics impose stricter requirements on segmentation models, particularly necessitating both robust global perception and precise local feature extraction capabilities.

To meet the precise segmentation requirements for transformer discharge carbon traces, this paper proposes the DCMC-UNet, a semantic segmentation model incorporating luminance adaptive enhancement and dynamic feature fusion. For resolving image blurring caused by unstable light reflection and supplemental lighting conditions in the transformer oil, we developed a luminance contrast adaptive enhancement algorithm that introduces learnable parameters to achieve balanced luminance and contrast in carbon trace defect images while simultaneously enhancing edge information.

Considering the morphological diversity and edge complexity of carbon traces, the encoder incorporates a dynamic deformable encoder (DDE) that optimizes feature extraction through deformable convolutional kernels. The decoder employs an edge-aware decoder (EAD) architecture that extracts boundary details from predicted segmentation maps and integrates them with encoded features to improve edge segmentation accuracy.

To bridge the semantic gap between the encoder and decoder, we replaced traditional skip connections with cross-level attention connection fusion layers (CLFCs), strengthening the cross-level integration of morphological and edge features. Furthermore, a multi-scale atrous feature aggregation module (MAFA) was designed in the neck network to enhance fusion and parsing of multi-dimensional features, enabling the effective aggregation of multi-scale defect characteristics. This design not only improves segmentation precision but also strengthens the model’s capability to identify carbon traces of varying sizes, significantly reducing the missed detection of small-scale carbon traces.

## 2. Transformer Internal Inspection Robot

### 2.1. Transformer Internal Inspection Robot Structure

**Figure 1:**
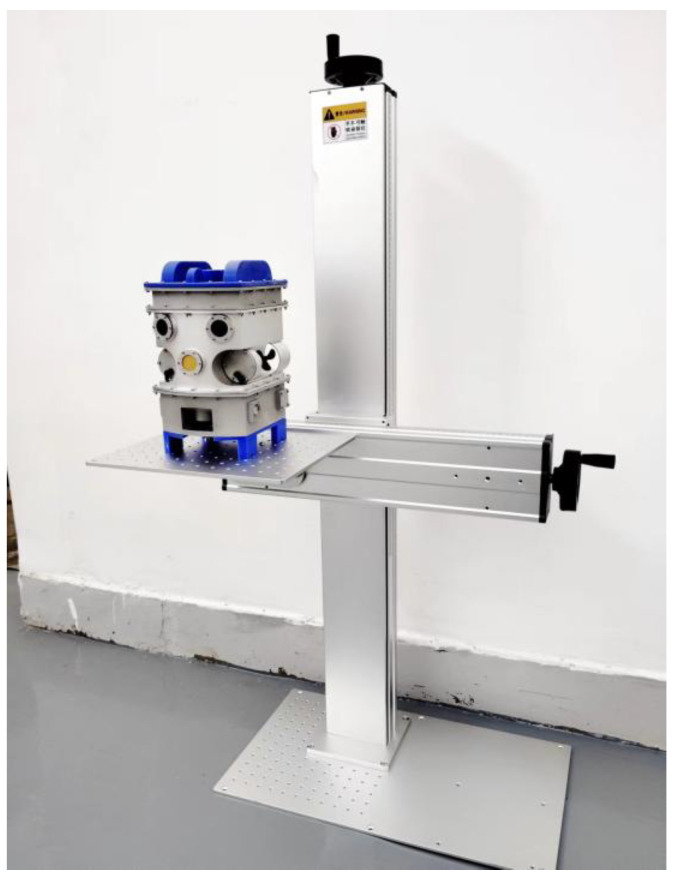
Overall structure of the transformer internal inspection robot.

The newly designed prototype of a next-generation internal inspection robot for transformers (structure shown in Figure 1) proposes an integrated solution to address the limitations of traditional inspection methods in the confined environment of oil-immersed transformers. This robotic system consists of core modules such as the inspection robot body, depth measurement device, omnidirectional positioning signal transmitter, dual-sided vision system, propulsion system, gravity balance device, infrared obstacle avoidance sensor, and control system, featuring multiple innovative design characteristics.

In terms of mobility, the robot adopts a streamlined body structure equipped with a bidirectional horizontal and vertical propulsion system. Optimized hydrodynamic design significantly reduces motion resistance, enhancing flexibility and stability in transformer oil media. Additionally, the innovative gravity balance adjustment device employs a dynamic buoyancy regulation mechanism to maintain an approximate balance between the robot’s weight and buoyancy. This not only substantially reduces energy consumption but also improves posture stability, extending the system’s operational endurance to 50 min.

The environmental perception system integrates high-precision ultrasonic positioning devices and infrared ranging sensors. By periodically emitting ultrasonic signals and coordinating with internal transformer positioning arrays, it achieves high-precision spatial positioning with a maximum positioning error of 20 mm and a relative positioning error within 3%. Combined with infrared obstacle avoidance, this ensures detection safety and operational reliability in complex environments.

As the core functional module, the visual inspection system employs a combination of a 30° wide-angle binocular camera on the front curved upper surface and a high-brightness, wide-angle spotlight in the center, effectively resolving imaging challenges in fully enclosed dark environments. A C3_ISC3NA high-definition industrial camera is centrally installed on the upper cover, utilizing H.265+ video compression technology to achieve 1080P ultra-low-bitrate image capture. Paired with LED fill lights on both sides, it ensures the clear imaging of insulation conditions. The accompanying MINI HOMER image transmission module has a compact size, low power consumption, and strong wall-penetration capability, supporting the synchronous transmission of three 1080P video streams via an 11 Mbps maximum bandwidth.

Furthermore, the current system achieves a continuous operation time of up to 50 min on battery power. The ground station is equipped with a 1S lithium battery, Type-C charging interface, and multiple network ports, providing comprehensive support for image acquisition and transmission during inspection tasks.

### 2.2. Transformer Internal Inspection Robot Operation Terminal

**Figure 2:**
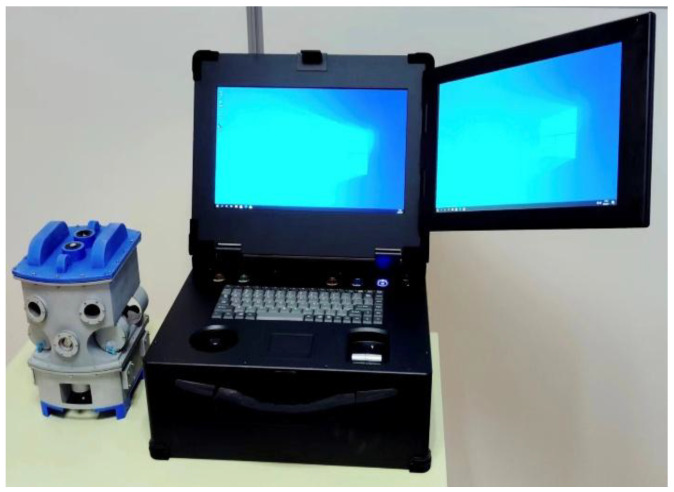
Operation terminal of the transformer internal inspection robot.

The operation terminal is a hardware device for monitoring the movement status of the internal inspection robot and receiving captured images of internal insulation, as shown in Figure 2. In addition, we are also equipped with portable control terminals, which are small in size and lightweight, making it easy for staff to carry them to the site for work.

The terminal consists of the following modules:

The Robot Operation Remote Control Module enables the precise manipulation of the robot’s vertical and horizontal movements including ascending, descending, forward, and backward navigation within the transformer oil; the Wireless Control Signal Communication Module establishes reliable transmission channels for the insulation images acquired by the robot; the 3D Spatial Positioning Software System Module provides the real-time localization of the robot’s spatial coordinates inside the transformer vessel, forming the foundation for operational decisions; and the Visual Defect Detection Module performs comprehensive image analysis to automatically identify and evaluate potential insulation degradation with detection algorithms.

The terminal-based transformer internal defect visual inspection system is developed in Python, featuring a GUI interface designed with the PySide6 framework. Its core functionalities include image preprocessing, carbon trace defect object detection, and instance segmentation, enabling the precise detection of discharge-induced carbon trace defects inside transformers to ensure equipment stability.

The software system builds the interactive interface through Qt Designer, which supports user login, model loading, image and video detection, and other functions. In the instance segmentation module, the user can select the pre-training model weights (.pt) and initialize the parameters, allowing the efficient segmentation of single or batch images, videos, and real-time camera-captured footage while accurately identifying and segmenting carbon trace defects. The system utilizes OpenCV to process image and video inputs, adopts multi-threading technology to optimize real-time segmentation efficiency, and visually presents segmentation results through mask annotation with statistical information (e.g., number of detected targets, elapsed time). The modular design of the operation terminal ensures ease of operation while significantly improving the scalability of the system, providing an efficient solution for the automated identification of internal transformer defects.

The transformer internal inspection robot integrates deep learning-based visual detection technology to achieve the precise identification and localization of carbon trace defects inside transformers through its high-precision vision system, thereby ensuring the safe operation of transformer equipment.

## 3. Construction of Transformer Discharge Carbon Trace Defect Dataset and Image Preprocessing

### 3.1. Transformer Discharge Carbon Trace Acquisition Experimental Platform

Carbon traces in transformers are formed through high-temperature arcing during partial discharge. When the local electric field intensity exceeds the insulation material’s breakdown threshold, arcing occurs and generates extreme heat. Sustained discharge causes thermal decomposition and carbonization of the insulation material, where organic components transform into carbon black or carbides under high temperatures, ultimately forming visible carbon traces. Based on this mechanism, we developed an experimental platform to simulate carbon trace formation and construct a defect sample library.

**Figure 3:**
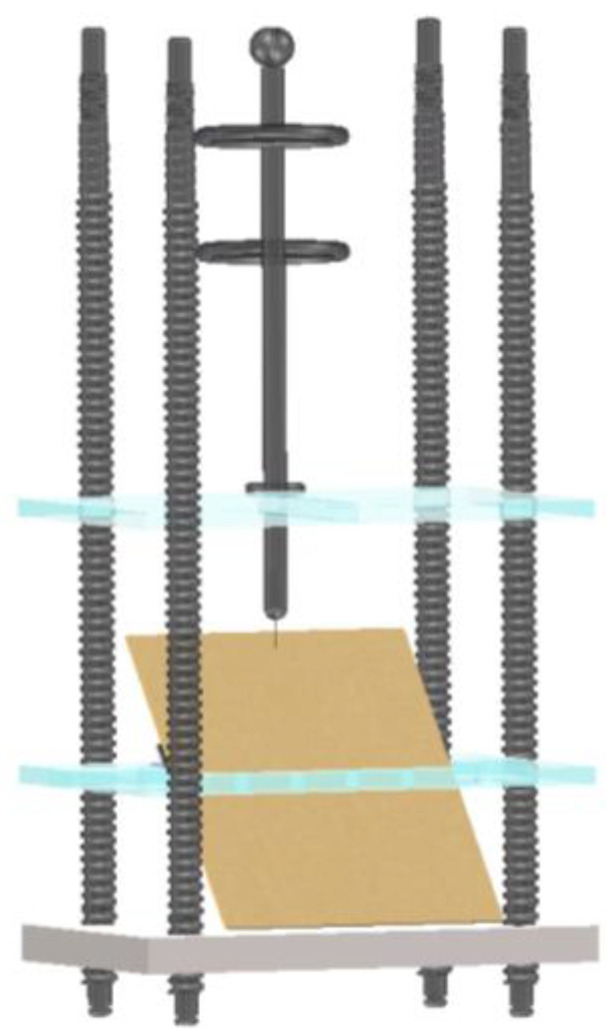
Needle–plate discharge model.

- The needle–plate model (structure shown in Figure 3):

Electrodes: A tungsten needle electrode with a 0.2 mm curvature radius, fixed by a brass front-end electrode.

Insulation Components: a 25 cm × 15 cm standard transformer pressboard, secured on an acrylic baseplate via an adjustable-angle nylon bracket.

Auxiliary Structures: nylon screws, grading rings, and connecting rods.

- Experimental Equipment:

Power System: SB-10KVA/100kV test transformer.

Observation System: HTSUA134GC/M high-speed camera (1.3 megapixels, 211 FPS).

Container Assembly: transparent acrylic test chamber (filled with Karamay No. 25 transformer oil).

**Figure 4:**
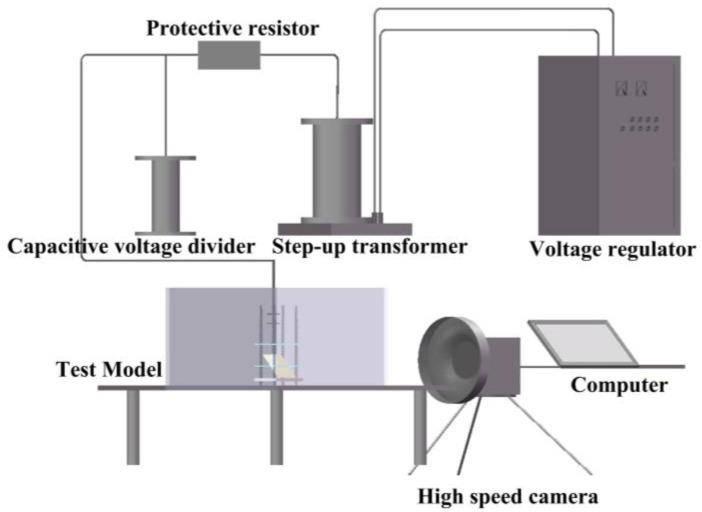
Wiring diagram of the experimental platform.

The specific electrical connection method of the experimental platform is shown in Figure 4. This design allows for the systematic study of carbon trace formation characteristics under different conditions by adjusting the pressboard inclination angle and discharge parameters.

Based on the aforementioned experimental setup, discharge simulation experiments were conducted. The high-speed imaging system was employed to capture the discharge process in real time, with the image data being simultaneously transmitted to a computer for observation and analysis.

A total of 654 typical discharge carbon trace defect images were ultimately obtained, comprising two categories: dendritic and clustered patterns. Due to the inherent difficulty in carbon trace formation, the number of samples collected through simulation experiments remained relatively limited. To expand the sample size, we initially performed a preliminary division of the sample library, selecting 206 images (116 dendritic and 90 clustered) as an independent test set. Subsequently, data augmentation through geometric transformations including rotation, mirroring, and stretching was applied to the remaining original samples, and a comprehensive sample library containing 3658 images (2329 dendritic and 1329 clustered images) was ultimately constructed.

**Figure 5:**
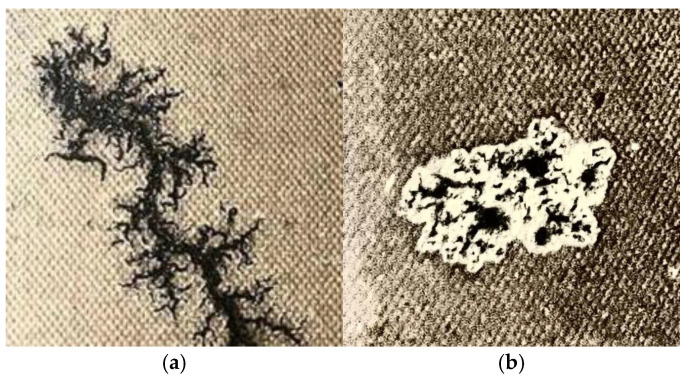
Transformer discharge carbon traces: (**a**) dendritic; (**b**) clustered.

The labeled images and labels were obtained after the carbon trace samples were labeled by LabelImg on a pixel-by-pixel, multi-point basis. This paper adopted a rigorous division scheme: maintaining the 206 original images as an independent test set, while randomly splitting the remaining images into training (2761 images) and validation (691 images) sets at an 8:2 ratio, thereby providing reliable data support for model training and evaluation. Representative carbon trace images are shown in Figure 5.

### 3.2. Luminance Contrast Adaptive Enhancement Algorithm

To address the challenges of insufficient illumination and complex background interference in images captured under the typically dark and complex acquisition environments inside transformers, this paper proposes a luminance contrast adaptive enhancement algorithm for preprocessing acquired carbon trace defect images. The proposed algorithm significantly improves visual quality by enhancing local contrast, suppressing noise, and augmenting edge information. These characteristics provide high-quality input data for subsequent defect detection and segmentation tasks. The algorithm’s superior performance in handling complex backgrounds, uneven illumination, and microscopic defects makes it an ideal solution for transformer defect image enhancement.

Existing image enhancement methods for preprocessing carbon trace defect images in transformers still exhibit significant limitations. Traditional gamma correction can adjust the grayscale dynamic range through parameter γ, yet its fixed parameter mechanism struggles to adapt to complex scenarios and may even reduce contrast under specific conditions [20,21,22]. While improved adaptive gamma correction methods introduce dynamic parameter adjustment, they often suffer from an over-enhancement of bright areas and insufficient improvement in low-illumination regions [23,24,25]. The CLAHE algorithm, though capable of noise suppression while enhancing contrast, still demonstrates inadequate adaptability when handling complex industrial environments such as transformer oil conditions [26].

Aiming at the limitations of traditional image enhancement methods in transformer oil-immersed environments, this paper proposes a brightness-contrast adaptive enhancement algorithm. The algorithm is based on the HSV color space for processing, and combines the optimization strategy of CLAHE and adaptive histogram weighted distribution gamma correction to achieve the adaptive enhancement of luminance contrast in complex oil-immersed environments, and at the same time avoids the problems of noise amplification and over-enhancement that exist in traditional algorithms.

When dealing with transformer internal defect images, since the red, green, and blue (RGB) images acquired by the camera have inter-channel interference and the lighting conditions are not directly related to a single channel, the algorithm in this paper firstly converts the RGB images into hue, saturation, and luminance (HSV) images. The three channels of the HSV images (H, S, and V) are independent of each other, in which the luminance (V) channel is directly related to the lighting conditions, and this conversion simplifies the lighting conditions. The three channels (H, S, and V) of the HSV images are independent of each other, and the luminance (V) channel is directly related to the lighting conditions, which simplifies the lighting correction operation and reduces the complexity of subsequent processing.

Subsequently, for the enhancement of the luminance (V) channel, this paper adopts the optimization strategy combining CLAHE and adaptive histogram weighted distribution gamma correction. This method can not only effectively improve the image brightness contrast but also avoid the common over-enhancement problem of traditional methods.

The algorithm is based on CLAHE for V-channel processing. It divides the image into a number of sub-regions and performs histogram equalization operation independently for each sub-region. To control the enhancement intensity, a contrast-limiting (clip limit) mechanism is introduced, which appropriately clips the gray histogram of each sub-region, confining its distribution within a reasonable range. However, in the complex environment inside the transformer, this histogram cropping process may amplify the background noise or lead to the over-enhancement of localized regions while improving the contrast of carbon trace defects. For this reason, we further introduce the adaptive gamma correction technique. This technique optimizes the image by dynamically adjusting the mapping relationship of pixel luminance values. Different from traditional fixed-parameter gamma correction, this method can automatically adjust the range of gray values according to the image histogram characteristics, ensuring the enhancement effect while maximizing the retention of useful details, thus significantly improving the overall image processing quality. The algorithm expression formula is as follows:

(1) $$G(x,y)=255{\left(\frac{X(x,y)}{255}\right)}^{\gamma }$$

where image X(x,y) is the image corrected by the CLAHE algorithm.

The adaptive gamma parameter γ is an equation with a weighted cumulative distribution function, as shown in the following equation:

(2) $$\gamma =1-cdf_{w}(i)$$

where $cdf_{w}(i)$ represents the weighted histogram cumulative distribution function.

The expression for the weighted histogram cumulative distribution function is as follows:

(3) $$cdf_{w}(i)=\sum_{i=0}^{X_{max}}\frac{p_{dfw}(i)}{\sum p_{dfw}}$$

where $X_{max}$ is the maximum gray value, $p_{dfw}(i)$ is the weighted histogram distribution function, and $\sum p_{dfw}$ is the weighted probability density function.

The expressions for the above functions are as follows:

(4) $$p_{dfw}(i)=p_{dfwmax}{\left(\frac{p_{df}(i)-p_{dfmin}}{p_{dfmax}-p_{dfmin}}\right)}^{\beta }$$

(5) $$\sum p_{dfw}=\sum_{i=0}^{X_{max}}p_{dfw}(i)$$

where β is the adjustment control parameter, and $p_{dfmax}$ and $p_{dfmin}$ are the maximum probability density and minimum probability density of the contrast-limited adaptive histogram, respectively.

The probability density function of an image X(x,y) can be expressed as follows:

(6) $$p_{df}(i)=\frac{n_{i}}{N}$$

where the gray value $n_{i}$ is the number of pixels of i, and N is the total number of pixels.

After completing the enhancement process for the V channel, the H, S, and V channels are merged and converted back to BGR space to obtain the final enhanced image.

### 3.3. Verification of Enhancement Effect of Transformer Internal Carbon Mark Defect Image

**Figure 6:**
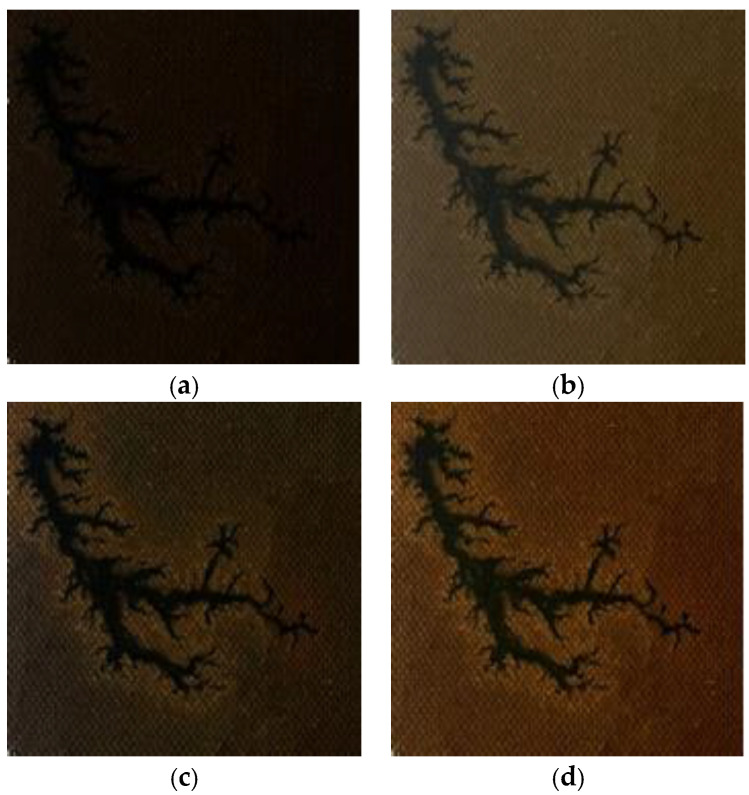
Comparison diagram of enhancement effects: (**a**) original image; (**b**) normal illumination; (**c**) CLAHE; (**d**) proposed algorithm.

This study validated the effectiveness of the proposed luminance contrast adaptive enhancement algorithm for improving carbon trace images of internal defects in oil-immersed transformers through comparative experiments. The experiments utilize dendritic discharge trace images captured under dark working conditions as test samples, with the CLAHE algorithm serving as the benchmark reference method. The assessment is further supplemented by a comparison with reference images obtained under normal lighting conditions. A comparison of the enhancement effects is illustrated in Figure 6.

From the comparison results in Figure 6, it can be seen that the improved algorithm proposed in this paper shows obvious advantages in terms of the image enhancement effect. Compared with the traditional CLAHE method, the improved algorithm achieves a better balance between brightness enhancement and contrast adjustment, and the image processed by the improved algorithm is closer to the defective reference image taken under normal lighting conditions in terms of the overall visual effect, which indicates that the algorithm successfully simulates the imaging characteristics under ideal lighting conditions.

**Figure 7:**
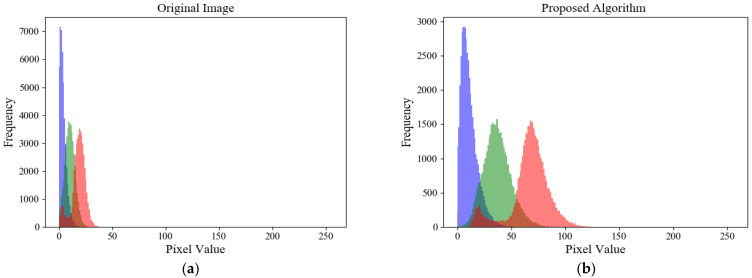
Comparison of image histograms: (**a**) histogram of the original image; (**b**) histogram of the enhanced image.

To further substantiate the algorithm’s efficacy, this study presents a comparative analysis of the histogram distributions between the original images and those processed by our proposed enhancement algorithm, as illustrated in Figure 7.

It can be observed from Figure 7a that the histogram of the original image exhibits a typical left-skewed unimodal distribution, with pixels concentrated in the dark range of [0, 50]. This implies that the original image has an excessively low brightness and the details in the dark areas are lost, making it impossible to directly observe the image features visually with the naked eye. The image processed by the luminance contrast adaptive enhancement algorithm proposed in this paper shows that the overly high peak in the histogram of the original image is reduced, the histogram distribution becomes uniform, and the histogram of the dark area expands to the right. This indicates that the brightness of the enhanced image has increased and the details in the dark areas have been restored.

In summary, the luminance contrast adaptive enhancement algorithm proposed in this study demonstrates substantial advantages in the image processing of internal defect traces within transformers. In comparison to traditional enhancement methods, the proposed algorithm exhibits superior performance in terms of brightness restoration and detail feature preservation, enabling the clear restoration of defect trace features. This provides a robust, high-quality image foundation for subsequent defect segmentation processes.

## 4. Improved U-Net-Based Discharge Carbon Trace Defect Segmentation Algorithm with Dynamic Feature Fusion

**Figure 8:**
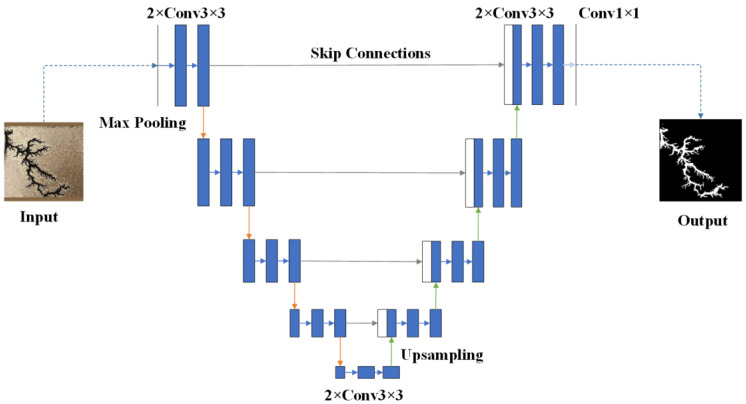
U-Net network architecture.

The U-Net architecture employs a characteristic encoder–decoder structure. The encoder progressively extracts features through convolutional and downsampling operations, utilizing max-pooling layers to reduce the spatial dimensions of feature maps. Each convolutional layer is followed by a ReLU activation function to enhance nonlinear representation capability. The decoder gradually restores feature map resolution through upsampling and convolutional operations. Skip connections concatenate feature maps from corresponding encoder and decoder levels to preserve spatial detail information. The network ultimately generates segmentation results with identical resolution to the input image. This architecture effectively captures contextual information through the encoder while recovering spatial details via the decoder, with skip connections enabling feature reuse to establish the fundamental image segmentation framework. The baseline U-Net network architecture is illustrated in Figure 8.

The visual data captured by internal transformer inspection robots is inherently constrained by the optical conditions within the equipment, resulting in prevalent low-luminance and non-uniform illumination issues. Moreover, the discharge carbon traces exhibit highly complex edge topology with significant scale variations. These characteristics render conventional U-Net algorithms markedly inadequate for carbon trace defect segmentation in transformers. Specifically, the fixed convolutional kernel structure in U-Net’s encoder fails to effectively capture the intricate features of carbon trace defects, leading to suboptimal feature extraction. The decoder, while competent in spatial dimension restoration, lacks targeted optimization for defect contours, resulting in poor boundary segmentation accuracy. Standard skip connections’ simplistic feature concatenation mechanism proves insufficient for reconstructing the complex edges of carbon traces. Furthermore, the limited receptive field in the neck network substantially compromises the extraction stability for defects of varying sizes.

**Figure 9:**
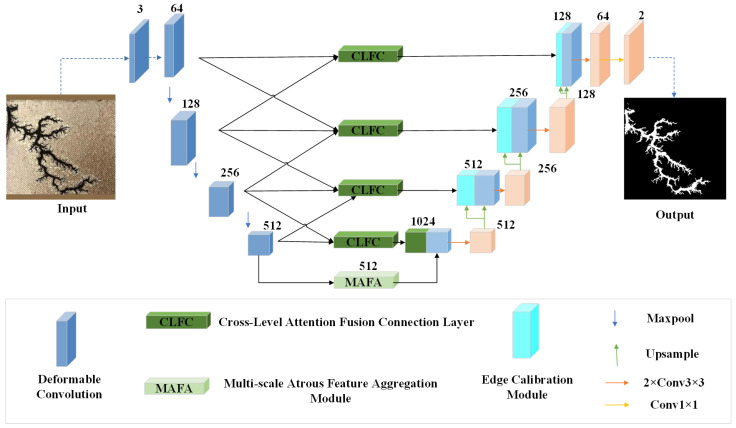
Improved network model architecture.

To address these challenges, this paper proposes an enhanced U-Net-based segmentation algorithm incorporating dynamic feature fusion for transformer discharge carbon trace defects. The improved network architecture, illustrated in Figure 9, introduces several key innovations specifically designed for the morphological diversity and edge complexity of carbon traces.

The encoder incorporates a dynamic deformable encoder (DDE) that adaptively adjusts convolutional kernel shapes to optimize feature extraction from variably-shaped carbon traces. In the decoder, the edge-aware decoder (EAD) explicitly extracts boundary details from predicted segmentation maps and integrates them with encoded features to enhance edge segmentation precision. Replacing traditional skip connections, cross-level attention connection fusion layers (CLFCs) are implemented to bridge the semantic gap between the encoder and decoder, significantly improving cross-layer fusion of carbon trace morphology and edge characteristics.

At the network neck, the multi-scale atrous feature aggregation module (MAFA) strengthens the integration of deep semantic features with shallow visual features through parallel multi-receptive-field processing. This hierarchical architecture achieves comprehensive fusion and parsing of multi-dimensional carbon trace features, enabling effective aggregation of defect characteristics across different scales. The combined improvements not only elevate segmentation accuracy but also enhance the model’s capability to identify defect traces of varying sizes, substantially reducing missed detections of microscopic carbon traces.

### 4.1. Enhanced Encoder–Decoder Architecture with Dynamic Feature Capture Mechanism

The standard U-Net encoder employs conventional convolution kernels with fixed geometric configurations, whose rigid receptive fields prove inadequate for accommodating the morphological diversity of carbon trace defects. This architectural limitation particularly compromises the network’s ability to capture irregular topological features such as branched patterns and crack formations. Furthermore, the max-pooling operations inherently cause positional information loss in deeper feature maps, exacerbating the localization inaccuracy. In the decoder pathway, traditional upsampling processes operate without explicit geometric constraints, focusing solely on spatial dimension restoration while neglecting targeted contour optimization. This fundamental design limitation manifests as reduced classification confidence for boundary pixels, ultimately impairing segmentation precision along defect edges.

To address these limitations in the encoder–decoder architecture, this paper proposes a Dynamic Feature Capture Mechanism (DFCM) that enhances adaptive feature extraction and fusion through targeted modifications to U-Net’s encoder–decoder pathways. The encoder incorporates a dynamic deformable encoder (DDE) module which introduces deformable convolutions to the base encoder structure. This implementation adds nine learnable offsets to standard 3 × 3 convolution kernels through a dynamic sampling mechanism, enabling the receptive field to adaptively expand according to defect morphology and thereby significantly improving the encoder’s feature extraction capability.

In the decoder pathway, the edge-aware decoder (EAD) integrates an Edge Calibration Module (ECM) that systematically extracts contour information from predicted segmentation maps at each network level. The EAD then fuses these boundary features with corresponding encoded features, ultimately outputting enhanced features optimized through edge-aware refinement. This DFCM approach achieves two critical improvements: (1) it strengthens the model’s feature extraction capacity through adaptive receptive field adjustment, and (2) implements an explicit boundary feature learning strategy that delivers remarkable precision gains for complex defect boundary segmentation tasks.

**Figure 10:**
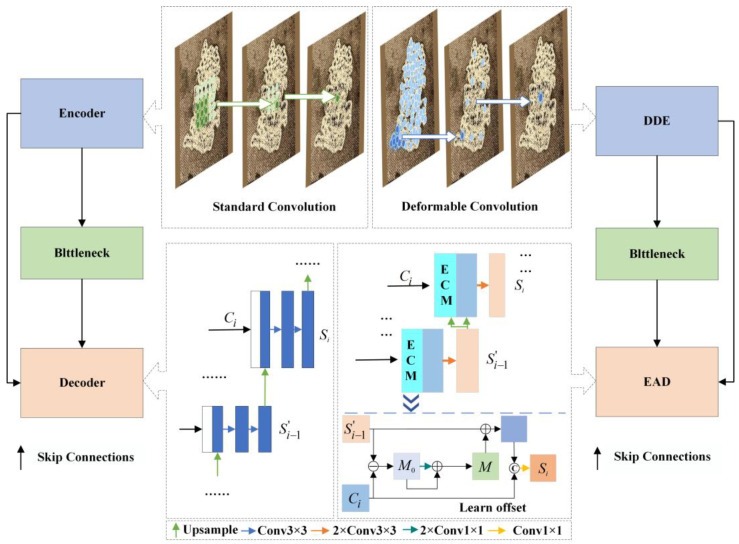
Encoder–decoder path: original vs. improved.

The architectural comparison between baseline and improved encoder–decoder pathways is illustrated in Figure 10.

The proposed dynamic deformable encoder (DDE) achieves adaptive feature extraction through dynamic adjustment of sampling positions, significantly enhancing the encoder network’s capability to model irregular features. The deformable convolution within the DDE dynamically modifies the sampling grid of convolution kernels, thereby better accommodating geometric deformations and irregular shapes of target objects [27]. This is implemented by introducing learnable offsets to each sampling point, enabling the convolution kernel to shift its sampling locations on the input image and concentrate on regions or objects of interest.

The mathematical formulation of the deformable convolution in DDE with offset introduction can be expressed as follows:

(7) $$y(p_{0})=\sum_{p_{n}\in R}w(p_{n})\times x(p_{0}+p_{n}+\Delta p_{n})$$

where $p_{n}$ represents the offset of each point in the convolution kernel relative to the center point, $\Delta p_{n}$ represents the offset, $w(p_{n})$ represents the weight of the convolution kernel in the corresponding position, $x(p_{0}+p_{n})$ represents the element value in position $p_{0}+p_{n}$ on the input feature map, and $y(p_{0})$ represents the element value in position $p_{0}$ on the output feature map. R represents the range of values for $p_{n}$: $R=\left\{\left(-1,-1\right),\left(-1,0\right),\ldots ,\left(0,0\right),\ldots ,\left(1,0\right),\ldots \left(1,1\right)\right\}$.

After introducing offsets, the sampling positions often yield fractional coordinates that do not directly correspond to actual pixels in the input feature map. To address this, interpolation methods are required to compute pixel values at these offset locations. In practical implementations, bilinear interpolation is the most commonly employed approach. This method calculates the interpolated pixel value as a weighted sum of its four nearest neighboring pixels (the closest valid points existing on the feature map), with the weights determined by the relative horizontal and vertical distances between the interpolation point and these neighboring pixels. The bilinear interpolation can be mathematically formulated as follows:

(8) $$\begin{array}{l} x(p)=\sum_{q}G(q,p)\times x(q)\\ =\sum_{q}g(q_{x},p_{x})\times g(q_{y},p_{y})\times x(q)\\ =\sum_{q}max(0,1-\left|q_{x}-p_{x}\right|)\times max(0,1-\left|q_{y}-p_{y}\right|)\times x(q) \end{array}$$

where q is the reference point, G represents the weight, $q_{x}$ is the x-coordinate of the reference point, $q_{y}$ is the y-coordinate of the reference point, $p_{x}$ is the x-coordinate of the pixel point, $p_{y}$ is the y-coordinate of the pixel point, and max(0,1−…) means that the distance between the interpolation point and the neighborhood point is limited to no more than 1 pixel. Finally, N feature maps are formed, and N convolution kernels are used to perform convolution operations, respectively, to obtain the output results.

To further enhance the module’s feature aggregation capability for target regions, we introduce a modulation mechanism on this basis. By controlling the offset weights of sampling points, the model can strengthen its focus on valid features within target areas while suppressing interference from background regions. Specifically, this mechanism learns a modulation parameter for each sampling point location, assigning higher weights to key features in target regions while reducing weights for irrelevant areas outside the targets, thereby achieving precise focus on regions of interest. The mathematical expression of this process is as follows:

(9) $$y(p_{0})=\sum_{p_{n}\in R}w(p_{n})\times x(p_{0}+p_{n}+\Delta p_{n})\times \Delta m_{k}$$

**Figure 11:**
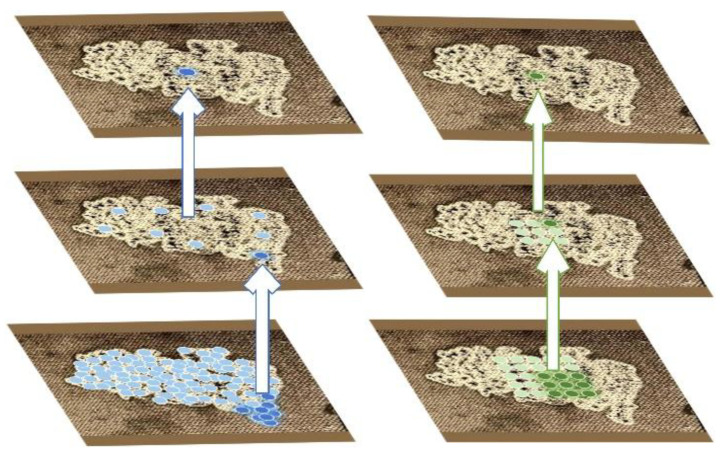
Convolution operations in conventional encoder vs. dynamic deformable encoder.

The proposed dynamic deformable encoder (DDE) effectively addresses the limitation of conventional encoders where fixed sampling positions in standard convolutions fail to adapt to target deformations, rotations, or irregular shapes. By incorporating learnable offsets, the DDE dynamically adjusts the sampling positions of convolutional kernels, enabling the convolution operation to adaptively conform to the geometric characteristics of targets. Figure 11 shows the workflow of the improved encoder convolution and the traditional encoder convolution.

**Figure 12:**
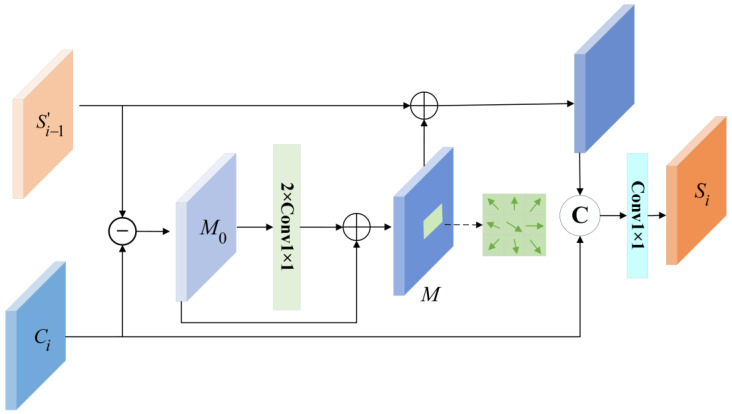
Schematic diagram of single-layer output in EAD.

In the decoder pathway, this paper proposes an edge-aware decoder (EAD) incorporating an Edge Calibration Module (ECM) to enhance the model’s capability in segmenting complex defect boundaries. As illustrated in Figure 12 for the l-th layer’s operation, the EAD systematically extracts edge details from predicted segmentation maps at each network level and subsequently integrates these boundary features with corresponding encoded features to generate edge-refined enhanced features [28].

For the edge calibration at level i, two inputs, $C_{i}$ and $S_{i-1}^{\prime }$, were used. $S_{i-1}^{\prime }$ is the prediction output of the previous network layer, and $C_{i}$ represents the encoded features produced by the decoder at the same resolution level. To generate the segmentation offset, $S_{i-1}^{\prime }$ is first subtracted from the prediction $C_{i}$ to produce the initial offset $M_{0}$, and then a residual convolutional layer is applied to learn the detailed segmentation offset, as follows:

(10) $$M=Conv(Conv(M_{0}))+M_{0}$$

(11) $$M_{0}=C_{i}-Upsampling(S_{i-1})$$

To generate refined segmentation outputs at each resolution level, the learned offset M is incorporated back into the preliminary segmentation $S_{i-1}^{\prime }$ to provide corrective refinement. This process can be formally represented as follows:

(12) $$S_{i}=Conv(Concat(M+S_{i-1}^{\prime }),C_{i})$$

where Conv represents the convolution operation and Concat represents the concatenation of feature channels.

The proposed edge-aware decoder (EAD) systematically enhances segmentation precision for complex carbon trace boundaries through its edge calibration mechanism and hierarchical feature reconstruction.

### 4.2. Enhanced Skip Connections and Neck Network with Multi-Level Context Fusion

The standard U-Net employs simple feature concatenation in its skip connections, which neither resolves the semantic gap between deep encoder features and shallow decoder features nor prevents the direct propagation of low-level noise, consequently reducing segmentation accuracy. The neck network’s fixed 3 × 3 convolutional kernels have limited receptive fields. While continuous downsampling in the encoder expands the receptive field and reduces computational load, it inevitably leads to feature information loss, resulting in unstable extraction of defects at different scales.

To address these limitations in skip connections and the neck network, this paper proposes a Multi-level Context Fusion Mechanism (MCFC). This mechanism enhances feature fusion capabilities and improves robustness in small defect detection by modifying both components: For skip connections, we designed cross-level attention fusion connection layers (CLFCs) to replace traditional U-Net skip connections for information transfer. This significantly mitigates the semantic gap between encoder and decoder while improving the cross-layer fusion of carbon trace morphology and edge features [29]. In the neck network, we developed a multi-scale atrous feature aggregation module (MAFA). MAFA employs dilated convolutional pyramid pooling to further enhance the fusion of deep semantic features with shallow visual features, improving the analysis and aggregation of multi-dimensional carbon trace characteristics [30].

**Figure 13:**
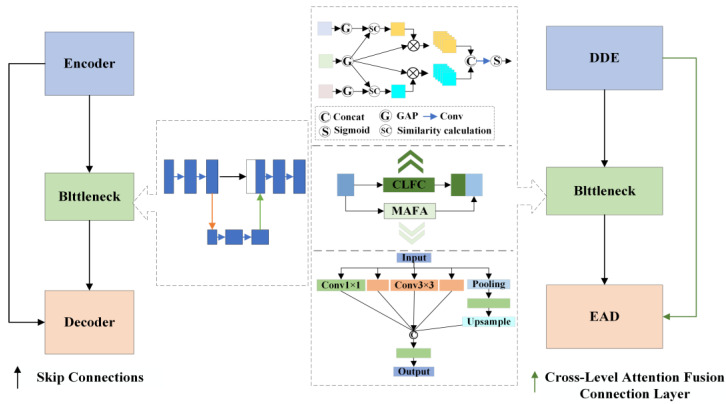
Skip connections and neck network: original vs. improved.

The MCFC not only improves segmentation accuracy but also enhances the model’s ability to recognize defect traces of different sizes, effectively reducing missed detection of microscopic carbon traces. Figure 13 shows the skip connections and neck network before and after improvement.

The proposed CLFC establishes cross-layer feature dependencies between the encoder and decoder, achieving dynamic fusion and enhancement of multi-level features. The CLFC leverages the large receptive fields and rich contextual information of high-level features to capture the overall morphological characteristics of targets while effectively integrating the precise spatial information provided by low-level features, ensuring accurate representation of local details.

Unlike simple feature concatenation, the CLFC intelligently selects and fuses multi-level features, which not only effectively resolves semantic discrepancies between the encoder and decoder but also significantly improves the transmission efficiency of carbon trace defect morphological features and the preservation of edge details, thereby optimizing the feature propagation path.

**Figure 14:**
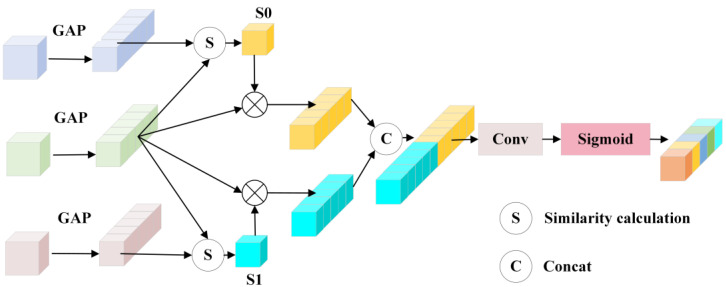
Detailed architecture diagram of the CLFC module.

A detailed schematic diagram of the proposed CLFC’s principle and structure is shown in Figure 14.

The figure shows that when CLFC transmits the information of layer i, it receives the features of the current layer and its two adjacent layers, namely, layer i−1 and layer i+1, which are denoted as $X_{i}$, $X_{i-1}$ and $X_{i+1}$, respectively. The CLFC first performs a global average pooling operation on $X_{i}$, $X_{i-1}$ and $X_{i+1}$ to generate three global vectors $\omega_{i}$, $\omega_{i-1}$ and $\omega_{i+1}\in {\mathbb{R}}^{C}$. Subsequently, $\omega_{i-1}^{clfc}$ is obtained by computing the inner product of $\omega_{i-1}$ with $\omega_{i}$ as the similarity score and multiplying this score with $\omega_{i}$. Similarly, $\omega_{i+1}^{clfc}$ is obtained, which is calculated as follows:

(13) $$\omega_{i-1}^{clfc}=(\omega_{i-1}⊙\omega_{i})\times \omega_{i}$$

(14) $$\omega_{i+1}^{clfc}=(\omega_{i+1}⊙\omega_{i})\times \omega_{i}$$

where ⊙ computes the similarity between two vectors.

Subsequently, $\omega_{i-1}^{clfc}$ and $\omega_{i+1}^{clfc}$ are concatenated and convolution is applied to generate ${\tilde{\omega }}_{i}^{clfc}$ with C channels using the following formula:

(15) $${\tilde{\omega }}_{i}^{clfc}=Conv(Concat(\omega_{i-1}^{clfc},\omega_{i+1}^{clfc}))$$

(16) $$X_{i}^{clfc}=X_{i}\cdot {\tilde{\omega }}_{i}^{clfc}$$

where Concat and Conv represent cascade and 3 × 3 convolutional layers, respectively, which are used to halve the channel dimension of the input tensor.

The CLFC performs feature recalibration by multiplying the input features $X_{i}$ with learned weights ${\tilde{\omega }}_{i}^{clfc}$. For the two outermost CLFC layers with only single input features, the individual cross-level features are directly fed into 3 × 3 convolutional layers without concatenation operations.

**Figure 15:**
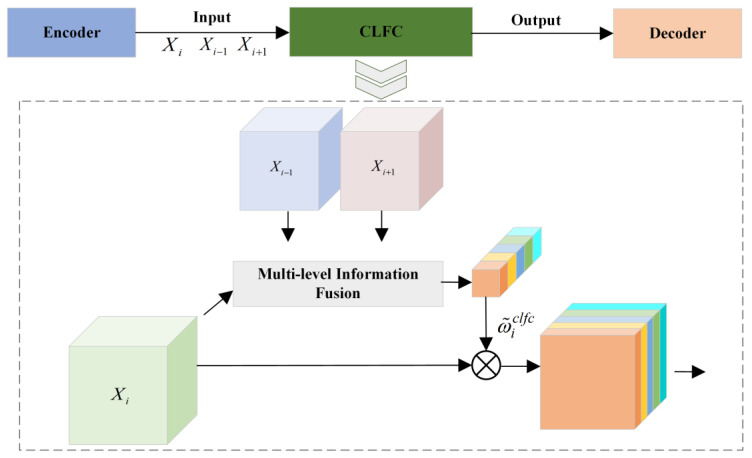
CLFC connection diagram.

This study proposes replacing the original skip connections in U-Net with cross-level attention fusion connection layers (CLFCs) for encoder–decoder information transfer and feature fusion, thereby optimizing the feature propagation path. A schematic diagram illustrating how the CLFC convolutional mechanism connects the encoder and decoder is shown in Figure 15.

**Figure 16:**
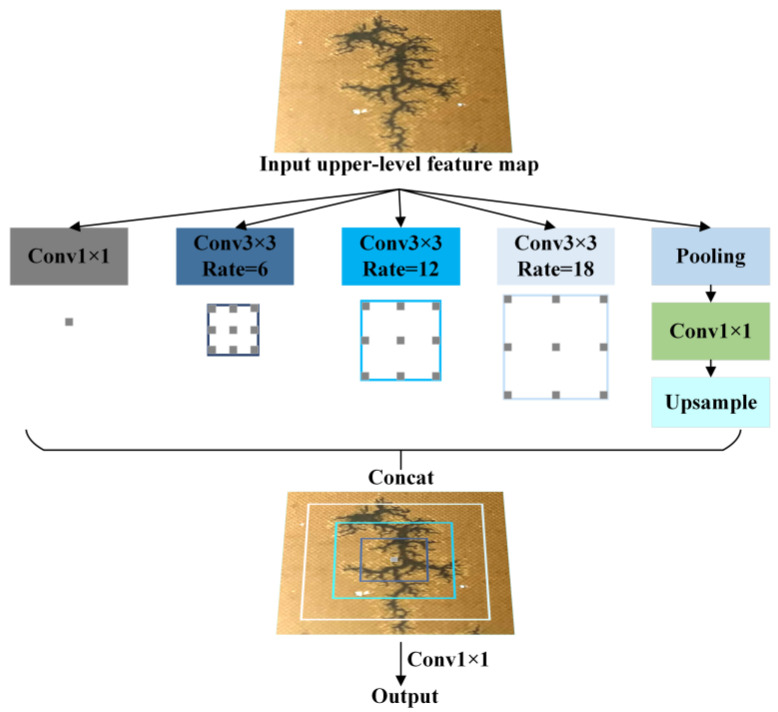
Schematic diagram of the MAFA structure.

In this study, the fourth downsampling layer and its corresponding convolutional module in the original U-Net were replaced with a multi-scale atrous feature aggregation module (MAFA). This modification reduces spatial information loss and enhances feature extraction efficiency for carbon trace images, thereby improving segmentation performance. The MAFA module employs dilated convolutions with varying sampling rates to extract multi-scale features, capturing richer contextual information. This multi-scale feature extraction mechanism not only effectively identifies features at different scales but also strengthens the model’s ability to analyze complex structures in carbon trace images through feature fusion. The MAFA architecture is illustrated in Figure 16.

The dilated convolution in MAFA introduces a dilation rate to expand the receptive field of the convolution kernel without adding extra parameters or computational costs. By increasing the spacing between the elements of the convolution kernel, it can cover a wider input area, effectively mitigating the loss of spatial resolution caused by downsampling. The MAFA module consists of four parallel branches: a standard 1 × 1 convolutional branch for capturing original-scale feature information., and three 3 × 3 dilated convolutional branches with varying dilation rates [6, 12, 18] to extract multi-scale contextual features. This design specifically addresses the morphological diversity of carbonized discharge traces in power transformers (e.g., the coexistence of slender dendritic structures and small clustered carbon traces). The selection of dilation rates [6, 12, 18] is based on the following: small dilation (6)—focuses on capturing fine edge details of micro carbon traces; medium dilation (12)—adapts to typical width variations of carbon tracks; large dilation (18)—covers abnormally wide or complex branching trace regions.

Appropriate padding is applied to maintain feature map dimensions post-convolution. All branches process the input feature maps in parallel, and their outputs are concatenated along the channel dimension. This enables the model to leverage multi-scale features simultaneously. The concatenated feature maps are then refined via a 1 × 1 convolution layer for integration and channel adjustment, followed by a Dropout layer to reduce overfitting risks and enhance generalization capability.

### 4.3. Loss Function

During model training, to ensure the edge alignment module learns offsets across different resolution levels constrained by both segmentation and boundary task losses, our method adopts a joint optimization strategy combining the boundary loss $\left(L_{edg}\right)$ with the composite segmentation loss $\left(L_{bce}+L_{dice}\right)$. The total loss function is formulated as follows:

(17) $$L_{loss}=\alpha (L_{bce}+L_{dice})+\beta L_{edg}$$

where α and β are the weights that balance the boundary loss and segmentation loss.

For the segmentation loss, $L_{bce}$ denotes the binary cross-entropy loss, $L_{dice}$ denotes the Dice loss, and the formulas are as follows.

(18) $$L_{bce}=-\sum_{i}(y_{i}\log{\hat{y}}_{i}+(1-y_{i})\log(1-\hat{y}))$$

(19) $$L_{dice}=1-\frac{2\left|y\cap \hat{y}\right|}{\left|y\right|+\left|\hat{y}\right|}$$

where y represents the true mask. $\hat{y}$ represents the prediction result.

For boundary loss, designed to dynamically optimize segmentation edges during training, it is defined as the binary cross-entropy between predicted edges and ground truth edges, expressed as follows:

(20) $$L_{edg}=L_{bce}(y_{b},{\hat{y}}_{b})$$

where $y_{b}$ represents the true mask. ${\hat{y}}_{b}$ represents the prediction result.

## 5. Validation of Model Performance Improvement and Result Analysis

### 5.1. Environment and Configuration

The experiment was conducted on the Windows 11 OS with an Intel Core i5-13400F CPU and NVIDIA GeForce 4060Ti GPU (16GB VRAM). The deep learning frameworks PyTorch 2.1.0 and Python 3.8 were employed. The training parameters included the following: a batch size of 8, initial learning rate of 1 × 10^−4^, input image dimensions of 640 × 640 pixels, and 200 training epochs.

### 5.2. Performance Evaluation Metrics

Standard image segmentation metrics were used: Precision (P), Recall (R), F1-score, Accuracy, Dice, and mean Intersection over Union (mIoU).

P and R are calculated by comparing segmentation results against ground truth labels, where P represents the proportion of true positives among all predicted positives, and R denotes the proportion of true positives among all actual positives. Their computational formulas areas follows:

(21) $$P=\frac{TP}{TP+FP}$$

(22) $$R=\frac{TP}{TP+FN}$$

where TP stands for true positive, FP stands for false positive, FN stands for false negative, and TN stands for true negative.

Accuracy measures the overall predictive correctness of the model across the entire image, representing the proportion of correctly predicted pixels relative to the total. It is calculated as follows:

(23) $$Accuracy=\frac{TP+TN}{TP+FN+TN+FP}$$

where TP stands for true positive, FP stands for false positive, FN stands for false negative, and TN stands for true negative.

To quantitatively assess the similarity between predicted segmentation regions and ground truth labels, two key metrics are employed: IoU and Dice.

The Intersection over Union (IoU) measures the overlap between predicted and ground truth regions, defined as the ratio of their intersection area to their union area:

(24) $$IoU=\frac{\left|A\cap B\right|}{\left|A\cup B\right|}$$

where A and B denote the ground truth and predicted regions, respectively.

mIoU computes the arithmetic mean of IoU values across all classes:

(25) $$mIoU=\frac{1}{N}\sum_{i=0}^{N}IoU_{i}$$

where N is the total number of instances and $IoU_{i}$ is the IoU of the ith instance.

Dice quantifies the similarity between predicted and ground truth regions, calculated as twice the intersection area divided by the total area of both regions:

(26) $$Dice=\frac{2\left|A\cap B\right|+\varepsilon }{\left|A\right|+\left|B\right|+\varepsilon }$$

where the adjustment degree set by ε to prevent the denominator from being 0 usually takes the value of $10^{-6}$.

### 5.3. Analysis of the Impact of Image Enhancement on Defect Segmentation Performance

In this paper, the effectiveness of our proposed luminance contrast adaptive enhancement algorithm is preliminarily verified through the subjective visual observation and histogram pixel output comparisons in Section 3.3. In order to further assess the impact of this algorithm on the performance of defect segmentation, this study adopts a more professional quantitative index for comprehensive analysis. Peak signal-to-noise ratio (PSNR), mean square error (MSE), structural similarity index (SSIM) and visual information fidelity (VIF) were selected to comprehensively evaluate the performance of the algorithm from the perspectives of noise suppression, pixel fidelity, structural preservation, and information completeness, respectively.

PSNR reflects the global difference between the enhanced image and the original image, with higher values indicating less distortion. MSE quantifies the pixel-level error, with lower values indicating less distortion introduced by the enhancement process. SSIM (range 0–1) evaluates the ability to maintain brightness, contrast, and structural features, and the closer the value of the index is to 1, the better the structural information is maintained and the higher the visual quality is in the enhanced image. VIF (range 0–1) measures the transmission fidelity of visual information, and a VIF value that is closer to 1 indicates that the loss of image information in the enhancement process is less and the enhancement quality is better. The performance of different image enhancement algorithms can be more comprehensively assessed by the above objective evaluation indexes.

The experiments compared the performance difference between the traditional CLAHE and the algorithm (Enhanced) in this paper, and the results are shown in Table 1.

According to the experimental results shown in Table 1, the luminance contrast adaptive enhancement algorithm proposed in this paper achieves significant improvement in all objective evaluation indexes. Compared with the CLAHE-processed image, the PSNR of this paper’s algorithm is improved from 27.57 to 28.45, the MSE is reduced from 113.63 to 84.89, the SSIM is improved from 0.74 to 0.86, and the VIF index is also improved from 0.93 to 0.96. These quantitative data fully prove that, compared with the traditional CLAHE method, the present algorithm not only controls noise amplification more effectively (PSNR increases) and introduces fewer aberrations (reduced MSE), but it also better preserves the structural features of the original image (SSIM increases) while maintaining the visual information more completely (the VIF is closer to 1). The algorithm avoids noise amplification and over-enhancement by introducing an adaptive gamma correction mechanism, which significantly improves the carbon trace brightness contrast and effectively preserves the carbon trace features while providing higher-quality input images for subsequent segmentation tasks.

**Figure 17:**
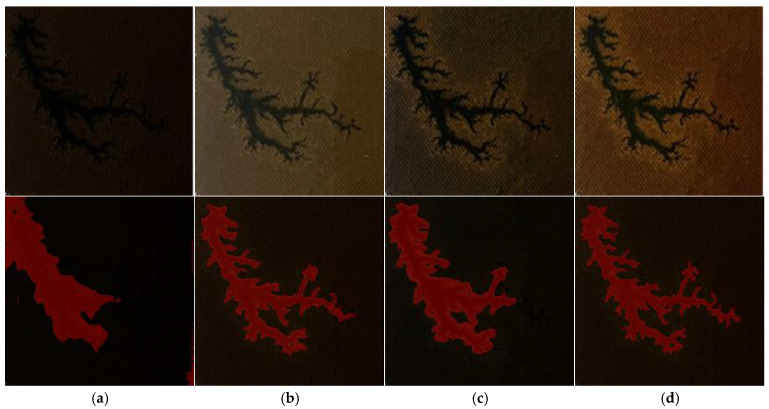
Segmentation result images: (**a**) original image; (**b**) normal illumination; (**c**) CLAHE; (**d**) proposed algorithm.

Subsequently, the DCMC-UNet segmentation model was employed to segment the original image, natural-light image, and images enhanced by different algorithms. The segmentation results are illustrated in Figure 17.

As shown in Figure 17, by comparing the segmentation results of images processed with different enhancement algorithms, the performance differences among these algorithms can be visually evaluated. The original dark-light image (shown in Figure 17a) has the least satisfactory segmentation effect due to the limitation of the lighting conditions, and there is obvious carbon trace feature omission detection; the traditional CLAHE algorithm (shown in Figure 17c) can extract the main carbon trace structure, but cannot perform the segmentation of the subtle branches and local defects, which indicates that it is deficient in detail retention; and the luminance contrast adaptive enhancement algorithm (shown in Figure 17d) proposed in this paper shows excellent performance, not only completely segmenting the complex topology of dendritic carbon traces but also accurately retaining the tiny feature details. In addition, the segmentation effect of the image enhanced by this algorithm not only significantly surpasses the original dark-light image and the CLAHE processing results, but it also surpasses the segmentation effect of the reference image under natural-light conditions. This result shows that the proposed algorithm achieves effective compensation for dark-light conditions and also further optimizes the degree of defective features in the image through adaptive adjustment. The enhancement technique reasonably enhances the brightness contrast and preserves the complete carbon trace features while strengthening the recognizability of the fine structure, which provides a better image base for the subsequent segmentation and diagnosis tasks.

### 5.4. Ablation Experiment

This paper proposes a U-Net-based algorithm for segmenting carbon trace defects in transformer discharges, incorporating dynamic feature fusion to address the morphological diversity and edge complexity of carbon trace defects. The encoder employs a dynamic deformable encoder, while the decoder integrates an edge-aware mechanism. To mitigate the semantic gap between the encoder and decoder, a cross-level attention fusion connection layer replaces the traditional skip connections in U-Net. Additionally, a multi-scale atrous feature aggregation (MAFA) module is implemented in the neck to enhance the fusion of deep semantic and shallow visual features.

To validate the rationality of the dilation rate selection in our proposed MAFA module, we conducted comparative experiments using multiple rate combinations—[1,2,3,4], [1,2,9,13], [1,3,6,12], and [1,6,12,24]—and compared these with our selected [1,6,12,18] configuration. The results are presented in Table 2.

As shown in Table 2, when comparing the Base model with five different dilation rate combinations ([1,2,3,4], [1,2,9,13], [1,3,6,12], [1,6,12,18], [1,6,12,24]), the [1,6,12,18] configuration demonstrates the most balanced and superior performance; it achieves 66.02% mIoU, outperforming the suboptimal [1,3,6,12] combination (64.54%) by 1.48 percentage points, and delivers the best Dice coefficient of 76.84%. It also maintains the highest Precision of 77.22%.

It is particularly noteworthy that using smaller dilation rates [1,2,3,4] leads to a significant 16.1 percentage-point drop in mIoU (from 64.51% to 48.41%). While the [1,6,12,24] configuration achieves the highest Accuracy (90.65%), its mIoU is actually 2.36 percentage points lower than that of [1,6,12,18]. Overall, the [1,6,12,18] dilation rate combination effectively avoids both the insufficient receptive field problem caused by small dilation rates and the excessive feature sparsity resulting from overly large dilation intervals, achieving the optimal balance between accuracy and feature coverage.

To evaluate the contribution of each improvement, we conducted comprehensive ablation experiments based on the baseline U-Net model. The experimental design progressively introduces the following modifications:

- Baseline Model (U-Net): The original structure without any enhancements.
- +DDE: Incorporates the deformable encoder.
- +DFCM: Integrates both the deformable encoder and edge-aware decoder.
- +DFCM+CLFC: Further replaces skip connections with the cross-level attention fusion layer.
- DCMC-UNet (improved model): Adds the MAFA module to the neck, completing all proposed improvements.

Performance was evaluated using Accuracy, Precision (P), mIoU, and the Dice coefficient.

As shown in Table 3, the results demonstrate the following:

1. Compared to the baseline U-Net, the +DDE model significantly improves all metrics, validating the enhanced feature extraction capability of the deformable encoder.
2. The +DFCM model (adding the EAD) further boosts performance across all indicators.
3. Introducing the CLFC layer (+DFCM+CLFC) leads to additional gains.
4. The complete DCMC-UNet (with MAFA) achieves optimal metrics, confirming the superiority of the proposed architecture.

This systematic ablation study highlights the cumulative benefits of each component in advancing segmentation accuracy and robustness for carbon trace defects.

Accuracy measures the proportion of correctly classified pixels relative to the total pixels. When comparing the baseline model with DCMC-UNet, their Accuracy values were 90.15% and 95.92%, respectively, showing a significant improvement of 5.77 percentage points. This demonstrates the model’s enhanced capability in overall pixel-wise classification accuracy.

Precision represents the ratio of true positive pixels among all predicted positive pixels. The baseline model achieved 74.71% Precision, while DCMC-UNet reached 85.68%, marking a substantial 10.97 percentage-point improvement. In the context of defect trace segmentation in transformers, this Precision enhancement indicates the model’s superior ability to accurately identify defective regions.

The mIoU metric evaluates the overlap between predicted and ground truth regions by calculating their Intersection over Union ratio. After implementing DFCM, the model’s mIoU improved from the baseline’s 64.60% to 74.83%, a 10.23 percentage-point gain, demonstrating the effectiveness of the proposed dynamic feature-capturing mechanism in improving shape and boundary segmentation accuracy. With all improvements implemented, mIoU further increased to 78.64%, showing continued performance enhancement.

The Dice coefficient measures the overlap between predicted and ground truth regions, providing an intuitive assessment of segmentation quality. The evaluation shows progressive improvements: the baseline U-Net achieved 76.07%, which increased to 80.61% after incorporating the deformable encoder, further improved to 83.63% with the edge-aware decoder, reached 85.37% after adding CLFC, and finally achieved 86.94% with all enhancements implemented.

These results clearly demonstrate consistent and significant performance improvements with each structural enhancement to the model. For more intuitive performance evaluation, we conducted a comparative analysis of segmentation outputs between the baseline model and our proposed dynamic feature fusion U-Net for transformer discharge carbon trace defect segmentation. The visual comparison in Figure 17 clearly illustrates the performance differences between these models.

**Figure 18:**
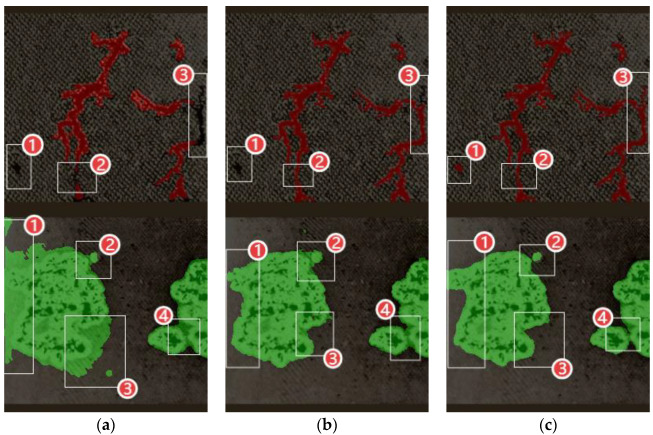
Comparative segmentation results between the baseline model and the proposed model: (**a**) baseline; (**b**) DFCM; (**c**) DCMC-UNet.

Figure 18 presents the following: (a) the defect image segmentation results from the baseline model (Baseline); (b) the segmentation results after incorporating the improved encoder–decoder structure (+DFCM); (c) the final segmentation results after implementing all enhancements (DCMC-UNet). In the figure, the red mask-covered area demonstrates that the region predicts the result of dendritic carbon traces, and the green mask-covered area demonstrates that the region predicts the result of clustered carbon traces.

Analysis of image (a) reveals that in dendritic trace segmentation, certain areas (marked by boxes ①–③) remain uncovered by the red mask, indicating missed detections. For clustered traces, obvious boundary inaccuracies are observed (boxed regions ①–④), where the baseline model incorrectly identified background as clustered carbon traces. The improved results in (b) show a significant reduction in both false and missed detections after encoder–decoder optimization: although minor missed detections persist in regions ① and ③ for dendritic traces, region ② achieves complete segmentation, with substantially reduced missed areas in region ③, while the clustered traces demonstrate remarkable refinement in all annotated regions (①–④). The fully optimized model in (c) exhibits outstanding performance, achieving the complete segmentation of dendritic traces without any missed detections and the precise edge extraction of clustered traces with zero false positives. These comparative results demonstrate that the DCMC-UNet model not only significantly improves segmentation accuracy for transformer discharge carbon traces, but it also achieves breakthrough progress in key aspects including complex edge extraction and reducing both false positives and missed detections.

**Figure 19:**
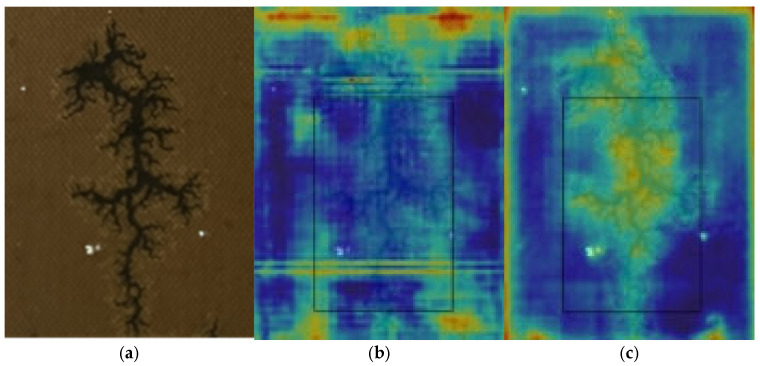
Comparison of heatmap outputs: (**a**) original image; (**b**) Baseline; (**c**) DDE.

We also compare the heatmap visualization effects before and after the decoder improvements in Figure 19. The results demonstrate the significant advantages of the proposed dynamic deformable encoder (DDE) in identifying dendritic carbon traces.

The heatmap visually demonstrates the model’s detection confidence distribution for carbon trace features through color gradients, where highlighted areas represent high-confidence locations identified by the model. As shown in the Figure 19, the Baseline’s heatmap exhibits significant flaws—its response regions are scattered and fail to accurately focus on actual carbon trace features, even displaying obvious misjudgments in non-carbon-trace areas. In contrast, the improved network shows remarkable performance enhancement. Its heatmap responses are precisely concentrated on genuine carbon trace regions, not only fully identifying the main trace paths but also achieving the high-precision localization of minor branch structures. This comparison thoroughly validates the effectiveness of the DDE network in feature-focusing capability.

### 5.5. Real-Time Performance Verification

In order to further verify the engineering significance of the DCMC-UNet segmentation model proposed in this paper, the optimal weights of the DCMC-UNet training were configured to the operation terminal of the transformer internal inspection robot to segment a single carbon trace image. This was implemented to verify the real-time performance of the model. The final results are shown in Table 4.

As can be seen from the table, the DCMC-UNet model demonstrates a significant computational efficiency advantage in terms of performance comparison on robotic devices. Although the FLOPs of the model reach 36.68 G, which is 3.28 G more than the baseline U-Net, and the number of parameters reaches 20.15 M, which is 2.29 M more, its inference speed reaches 76.31 FPS, which is 26.45 FPS more than that of U-Net. This improvement not only maintains the computational complexity of the model within the acceptable range, but, more importantly, it significantly improves the inference speed, so that it fully meets the real-time requirements of more than 30 FPS in practical engineering applications, especially suitable for application scenarios such as robot inspection, which has high requirements for real-time performance. The experimental results show that through the optimized design of the model structure, DCMC-UNet achieves more efficient inference performance while ensuring the reasonable consumption of computational resources.

### 5.6. Comparative Experiments with Different Models

To validate the effectiveness and advantages of our dynamic feature fusion U-Net for transformer discharge carbon trace defect segmentation, we conducted comparative experiments with PSPNet, DeepLabV3+, U2Net, UNet++, SegFormer, TransUNet, and UNet3+, these representing major architectural advances in segmentation with proven performance across various tasks. All models were trained and evaluated on our test set, with quantitative results shown in Table 5. The comprehensive comparison demonstrates our model’s superior capability in handling the specific challenges of transformer internal defect segmentation.

The experimental results in Table 5 clearly show that the DCMC-UNet model proposed in this paper significantly outperforms the other comparative models across all evaluation indexes. Specifically, the Accuracy of DCMC-UNet reaches 95.92%, which is 4.57 percentage points higher than that of the next-best-performing model, U-Net++. Precision is 85.68%, which is 1.75 percentage points higher than PSPNet’s 83.93%. The mIoU score of 78.64% represents an 11.35 percentage-point advantage over PSPNet. With a Dice of 86.94%, DCMC-UNet surpasses all other models, outperforming the second-ranked PSPNet by 8.59 percentage points. These quantitative metrics comprehensively validate DCMC-UNet’s superior performance in key evaluation indexes including Accuracy, Precision, mIoU, and Dice, demonstrating clear advantages over existing models such as DeepLabV3+, PSPNet, U2Net, U-Net++, UNet3, TransUNet, and SegFormer. The results conclusively prove DCMC-UNet’s outstanding capability in segmenting carbon trace defects within transformers.

**Figure 20:**
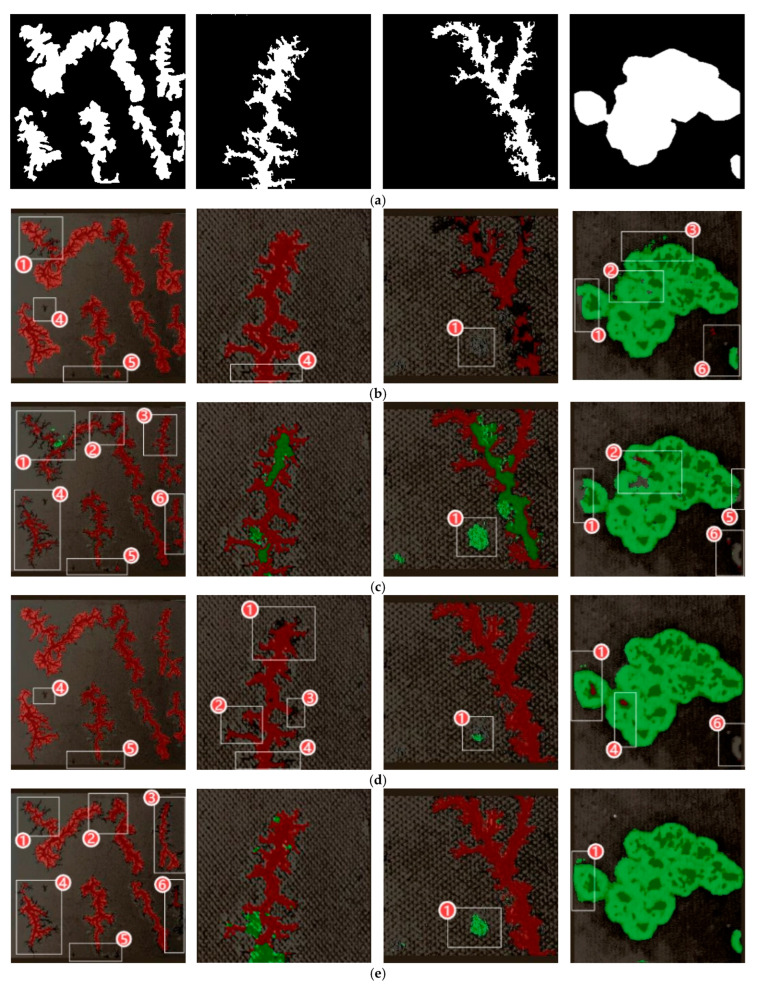

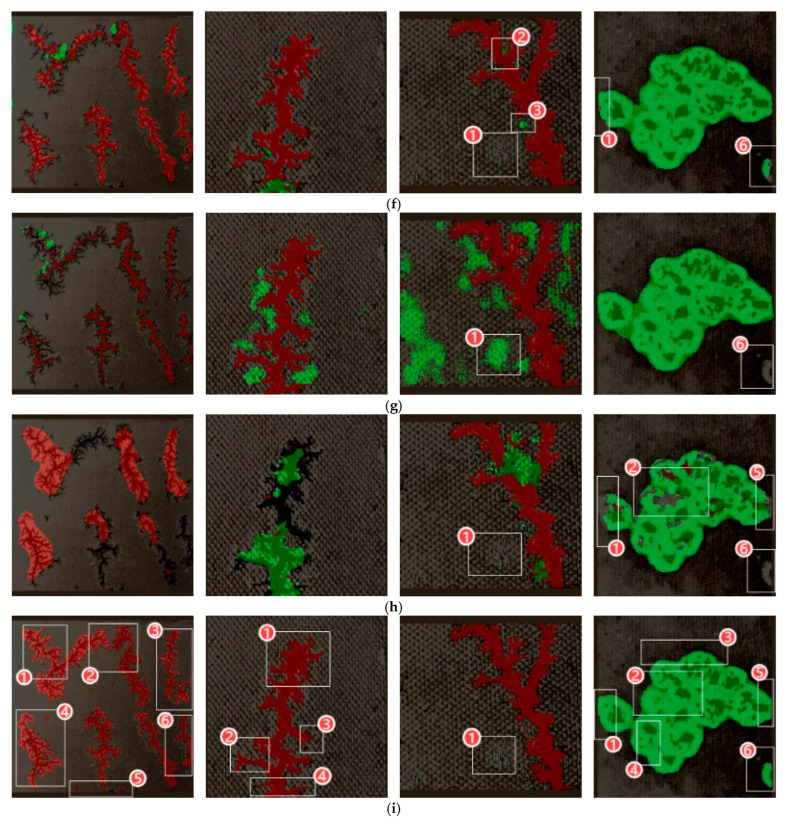
Comparison of segmentation results across models: (**a**) masks; (**b**) DeepLabV3+; (**c**) PSPNet; (**d**) U2Net; (**e**) U-Net++; (**f**) UNet3; (**g**) TransUNet; (**h**) SegFormer; (**i**) improved.

For visual comparison, Figure 20a presents the ground truth masks of carbon trace defects in transformers, while Figure 20b–i provide comparative segmentation results from different models.

The experimental results in Figure 20 clearly demonstrate DCMC-UNet’s superior segmentation performance compared to existing methods, with its output showing the closest visual agreement to ground truth labels. In the first test image, the conventional models exhibit significant shortcomings: those in Figure 20b–e fail to adequately segment small targets in regions ①–⑥, while those in Figure 20c,f,g display severe false positives by misclassifying dendritic carbon traces as clustered ones (incorrectly masked in green). Figure 20h shows incomplete coverage of dendritic traces (red mask), indicating missed detections. In contrast, DCMC-UNet alone successfully captured complete defect contours while preserving subtle details without omission.

Similar advantages can be observed in the second dendritic trace image, which shows that competing models (c) and (e)–(h) frequently misclassified dendritic patterns as clustered traces, revealing fundamental limitations in feature discrimination. While models (b) and (d) showed relatively better performance, they still failed to completely segment fine connecting branches in regions ①–④. Only DCMC-UNet (i) achieved complete and accurate reconstruction of the carbon trace morphology.

In the segmentation of the third dendritic carbon trace image, we observe that in the region labeled ①, models (b), (f), (h), and (i) avoid false positives. However, model (b) exhibits extensive missed detections, with its red mask failing to fully cover the dendritic structures. Models (h) and (f) show minor false detections in small areas. Notably, the DCMC-UNet model demonstrated perfect segmentation performance without any missed or false detections.

For the fourth clustered carbon trace segmentation task, only models (b), (e), and (i) successfully achieved the complete segmentation of the small carbon trace in the lower-right corner (marked as region ⑥). Comparing the segmentation results in regions ①–⑤ across all models, all other models except (i) exhibit minor instances of missed detections, false positives, and imprecise boundary segmentation.

From a comprehensive point of view, DCMC-UNet achieves the optimal segmentation effect in all the test cases, which can completely capture the target features and effectively avoid all kinds of misjudgment phenomena, fully demonstrating its stability and accuracy in complex scenes. The visual evidence systematically confirms DCMC-UNet’s significant advantages over existing approaches in handling challenging segmentation tasks involving complex carbon trace patterns.

## 6. Conclusions

To address the blurred defect images caused by unstable light reflection and supplementary illumination in transformer oil dielectric environments, this paper pro-poses an enhanced CLAHE algorithm optimized through the introduction of learnable parameters. The enhanced algorithm is capable of autonomously calibrating the brightness and contrast of carbon trace defect images, while concurrently amplifying the edge characteristics of dendritic carbon traces. The experimental findings demonstrate that the proposed algorithm not only more effectively controls noise amplification (the PSNR improved from 27.57 to 28.45) but also reduces distortion (the MSE decreased from 113.63 to 92.95). Furthermore, it better preserves the original image structure (SSIM = 0.86, which is closer to 1) and retains more complete visual deformation (VIF = 0.94, which is closer to 1).

To address the morphological complexity of carbon trace defects in transformers, we propose DCMC-UNet, integrating a dynamic deformable encoder (DDE) for adaptive feature extraction, an edge-aware decoder (EAD) for refined segmentation, a cross-level fusion connection (CLFC) to bridge semantic gaps, and a multi-scale atrous feature aggregation (MAFA) module for enhanced contextual learning. Compared to the baseline model, DCMC-UNet improves the mIoU by 14.04%, Dice by 10.87%, Precision by 10.97%, and Accuracy by 5.77%. It effectively addresses detection challenges caused by complex shapes, size variations, and irregular edges. This provides transformer inspection robots with reliable technical support for internal defect diagnosis.

## Figures and Tables

**Table 1:** Evaluation metric results of different algorithms.

Algorithms	PSNR	MSE	SSIM	VIF
CLAHE	27.57	113.63	0.74	0.93
Enhanced	28.45	92.95	0.86	0.94

**Table 2:** Comparative experiment on MAFA performance with different expansion rates.

Rate	Accuracy	P	mIoU	Dice
Base	89.02%	74.45%	64.51%	76.01%
[1,2,3,4]	88.03%	76.34%	48.41%	62.70%
[1,2,9,13]	90.40%	72.95%	63.25%	75.14%
[1,3,6,12]	89.28%	75.32%	64.54%	75.69%
[1,6,12,18]	89.62%	77.22%	66.02%	76.84%
[1,6,12,24]	90.65%	76.94%	63.66%	76.25%

**Table 3:** Ablation study results.

Model	Accuracy	P	mIoU	Dice
U-Net	90.15%	74.71%	64.60%	76.07%
+DDE	93.37%	82.05%	70.28%	80.61%
+DFCM	94.35%	83.63%	74.83%	83.63%
+DFCM+CLFC	95.10%	82.95%	76.98%	85.37%
DCMC-UNet	95.92%	85.68%	78.64%	86.94%

**Table 4:** Real-time performance verification.

Model	Time/s	FPS	GFLOPS	Params/M
U-Net	0.020	49.86	31.40	17.26
DCMC-UNet	0.013	76.31	34.68	20.15

**Table 5:** Comparison of experimental results of different models.

Model	Accuracy (%)	Precision (%)	mIoU (%)	Dice (%)
DeepLabV3+	86.99	82.93	58.90	71.79
PSPNet	89.21	83.93	67.29	78.35
U2Net	89.58	71.86	65.18	76.11
U-Net++	91.35	77.94	65.58	77.13
UNet3	89.77	67.69	65.00	76.12
TransUNet	88.58	63.97	61.19	72.93
SegFormer	89.54	73.38	54.57	68.36
DCMC-UNet	95.92	85.68	78.64	86.94

## Data Availability

The data presented in this study are available on request from the corresponding author. The data are not publicly available due to privacy.
